# Impact of Different Training Modalities on Anthropometric and Metabolic Characteristics in Overweight/Obese Subjects: A Systematic Review and Network Meta-Analysis

**DOI:** 10.1371/journal.pone.0082853

**Published:** 2013-12-17

**Authors:** Lukas Schwingshackl, Sofia Dias, Barbara Strasser, Georg Hoffmann

**Affiliations:** 1 Department of Nutritional Sciences, Faculty of Life Sciences, University of Vienna, Vienna, Austria; 2 School of Social and Community Medicine, University of Bristol, Bristol, United Kingdom; 3 Institute of Nutritional Sciences and Physiology, University for Health Sciences, Medical Informatics and Technology, Hall in Tirol, Austria; University of Bath, United Kingdom

## Abstract

**Background:**

The aim of this systematic review of randomized controlled trials was to compare the effects of aerobic training (AET), resistance training (RT), and combined aerobic and resistance training (CT) on anthropometric parameters, blood lipids, and cardiorespiratory fitness in overweight and obese subjects.

**Methods:**

Electronic searches for randomized controlled trials were performed in MEDLINE, EMBASE and the Cochrane Trial Register. Inclusion criteria were: Body Mass Index: ≥25 kg/m^2^, 19+ years of age, supervised exercise training, and a minimum intervention period of 8 weeks. Anthropometric outcomes, blood lipids, and cardiorespiratory fitness parameters were included. Pooled effects were calculated by inverse-variance random effect pairwise meta-analyses and Bayesian random effects network meta-analyses.

**Findings:**

15 trials enrolling 741 participants were included in the meta-analysis. Compared to RT, AET resulted in a significantly more pronounced reduction of body weight [mean differences (MD): -1.15 kg, p = 0.04], waist circumference [MD: -1.10 cm, p = 0.004], and fat mass [MD: -1.15 kg, p = 0.001] respectively. RT was more effective than AET in improving lean body mass [MD: 1.26 kg, p<0.00001]. When comparing CT with RT, MD in change of body weight [MD: -2.03 kg, p<0.0001], waist circumference [MD: -1.57 cm, p = 0.0002], and fat mass [MD: -1.88 kg, p<0.00001] were all in favor of CT. Results from the network meta-analyses confirmed these findings.

**Conclusion:**

Evidence from both pairwise and network meta-analyses suggests that CT is the most efficacious means to reduce anthropometric outcomes and should be recommended in the prevention and treatment of overweight, and obesity whenever possible.

## Background

Recent data provided by the World Health Organization illustrates that the global prevalence of overweight and obesity has more than doubled since 1980. In 2008, more than 1.4 billion adults aged 20 and older were overweight with over 500 million of them being obese [1]. Since overweight and obesity are evidence-based risk factors for diabetes, cardiovascular disease (CVD) or cancer, [2] the emergence of obesity as a pandemic represents a serious challenge for public health authorities. Exercise and diet are established cornerstones in the primary prevention as well as in the management of obesity [3]. Thus, the American College of Sports Medicine and the American Heart Association both recommend either moderate-intensity aerobic physical activity for a minimum of 30 min on five days each week or vigorous-intensity aerobic activity for a minimum of 20 min on three days each week for healthy adults. In addition, these authorities encourage regular resistance training (RT) for a minimum of two days per week performing 8 exercises with 8–12 repetitions [4].

Several meta-analyses investigated the independent effects of aerobic exercise training (AET) on anthropometric and cardio-metabolic risk factors, providing evidence for reductions in body mass index (BMI), body weight (BW), waist circumference (WC), visceral adipose tissue, and increasing high-density lipoprotein cholesterol (HDL-C) and maximum oxygen uptake (VO_2_max) [5]–[9]. With respect to RT, some meta-analyses reported reductions of HbA1c, systolic blood pressure (SBP), diastolic blood pressure (DBP), and C-reactive protein (CRP) [10]–[12].

The above mentioned meta-analyses were performed in order to compare one or more of the training modalities with the data from a sedentary control group. To date, no systematic review has pooled the effects of different training modalities on anthropometrical and cardiovascular risk factors. Therefore, the aim of the present study was to conduct a systematic review and meta-analysis of randomized controlled trials (RCTs) to assess the efficacy of AET, RT, and CT on anthropometric outcomes, blood lipids, and cardiorespiratory fitness in overweight and obese subjects.

## Methods

The review protocol was registered in PROSPERO International Prospective Register of Systematic Reviews (crd.york.ac.uk/prospero/index.asp Identifier: CRD42013003905).

### Literature Search

Queries of literature were performed using the electronic databases MEDLINE (between 1966 and December 2012), EMBASE (between 1980 and December 2012), and the Cochrane Trial Register (until December 2012) restricted to randomized controlled trials and quasi-randomized controlled trials, but no restrictions to calendar date. The following search terms were used: (*“strength* AND *training”; “resistance* AND *training”; “aerobic* AND *training”; exercise* AND *training; endurance* AND *training*). Moreover, the reference lists from retrieved articles, systematic reviews, and meta-analyses were checked to search for further relevant studies. This systematic review was planned, conducted, and reported in adherence to standards of quality for reporting meta-analyses [13]. Literature search was conducted independently by two authors, with disagreements resolved by consensus.

### Eligibility Criteria

Studies were included in the meta-analysis if they met all of the following criteria: *(1)* randomized controlled design; *(2)* minimum intervention period of 8 weeks; *(3)* body mass index ≥25 kg/m^2^; *(4)* age: ≥19 years; *(5)* comparison of either AET vs. RT and/or CT vs. AET and/or CT vs. RT; *(6)* assessment of “primary outcome” markers: BW, WC, waist to hip ratio (WHR), fat mass (FM, given in kg), lean body mass (LBM, given in kg); assessment of “secondary outcome” markers: total cholesterol (TC), low-density lipoprotein cholesterol (LDL-C), HDL-C, triacylglycerols (TG) and VO_2_ max; *(7)* report of post-treatment mean values (if not available mean of changes from baseline were used) with standard deviation (or data suitable to calculate these parameters: standard error, 95% confidence interval); *(8)* training had to be supervised, not home-based; *(9)* exclusion of studies with a dietary co-intervention that was not applied in all intervention groups; *(10)* exclusion of subjects with type 2 diabetes, and coronary heart disease.

All abstracts and full text were assessed for eligibility independently by two authors.

### Risk of Bias Assessment

**Figure 1:**
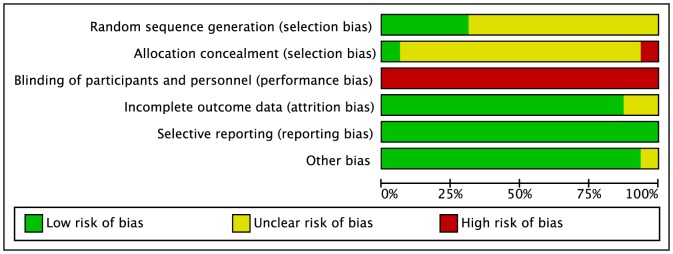
Risk of bias assessment tool. Across trials (n = 15), information is either from trials at a low risk of bias (green), or from trials at unclear risk of bias (yellow), or from trials at high risk of bias (red).

Full copies of studies were independently assessed by two authors for methodological quality using the risk of bias assessment tool by the Cochrane Collaboration [14], [15]. The following sources of bias were detected: selection bias (random sequence generation, allocation concealment), performance/detection bias (blinding of outcome assessment), attrition bias (incomplete data outcome), reporting bias (selective reporting) and other bias (in this case, trials were assessed for risk of bias in relation to “systematic difference in care”) (Figure 1).

### Data Extraction and statistical analysis

The following data were extracted from each study: the first author's last name, publication year, study duration, participant's sex and age, BMI, sample size, treatment effects, intervention type, dose, intensity and frequency, post mean values or differences in mean of two time point values with corresponding standard deviation. For each outcome measure of interest, pairwise and network random-effects meta-analyses were performed in order to determine the pooled relative effect of each intervention relative to every other, in terms of mean differences (MDs) between the post-intervention (or change from baseline differences in means) values of the different interventions. Combining both the post-intervention values and difference in means in one meta-analysis is a legitimate method described by the Cochrane Collaboration [15], which assumes that the relative effects estimated by both the post-intervention and the mean change from baseline measures is the same. However an additional assessment of baseline comparability was done and is summarized in Table S2 in File S1. Data were pooled if outcomes were reported by at least three studies. Heterogeneity between trial results was tested with a Cochran's Q test. A value for I^2^>50% was considered to represent substantial heterogeneity [16]. To consider heterogeneity, the random-effects model was used to estimate MDs with 95% confidence intervals (CIs). Forest plots were generated to illustrate the study-specific effect sizes along with a 95% CI. To determine the presence of publication bias, the symmetry of the funnel plots in which mean differences were plotted against their corresponding standard errors were assessed. Additionally, Begg's and Egger regression tests were performed to detect small study effects [17], [18].

Pooled effect sizes from the network meta-analyses are presented as posterior medians and 95% credible intervals (CrI) (i.e. Bayesian equivalent of confidence intervals) in the appropriate units. Separate pairwise meta-analyses were first used to compare all interventions. Network meta-analysis was then used to synthesize all the available evidence [19]. Network meta-analysis methods are extensions of the standard pairwise meta-analysis model which enable simultaneous comparison of multiple interventions whilst preserving the internal randomization of individual trials. They have the advantage of adequately accounting for the correlation in relative effect estimates from 3-arm trials as well as providing a single coherent summary of all the evidence. For pairwise meta-analyses, data were analyzed using the REVIEW MANAGER 5.1 software, provided by the Cochrane Collaboration (http://ims.cochrane.org/revman). Network meta-analyses were conducted using Markov chain Monte Carlo (MCMC) simulation implemented through the freely available software WINBUGS, version 1.4.3 [20]. The WINBUGS code used is freely available online [19], [21] (program “TSD2-5aRE_Normal_id.odc”).

Minimally informative normal priors were used for all treatment effect parameters and a Uniform (0,150) prior was used for the between-study standard deviation (heterogeneity) parameter. Sensitivity to this prior was assessed, but there was no meaningful change in relative effects or overall conclusions.

Three MCMC chains were used to assess convergence using Brooks-Gelman-Rubin plots and by inspection of the trace plots [22]. Convergence was achieved after 20,000 iterations for all outcomes. Posterior summaries were then obtained from further simulation of 50,000 iterations in each of the 3 chains (150,000 in total), resulting in a small Monte Carlo error.

The potential for inconsistency was assessed by inspection of the available evidence. In case of possible inconsistency, Bayesian p-values for the difference between direct and indirect evidence were calculated, and direct and indirect estimates were compared [23], [24].

## Results

**Figure 2:**
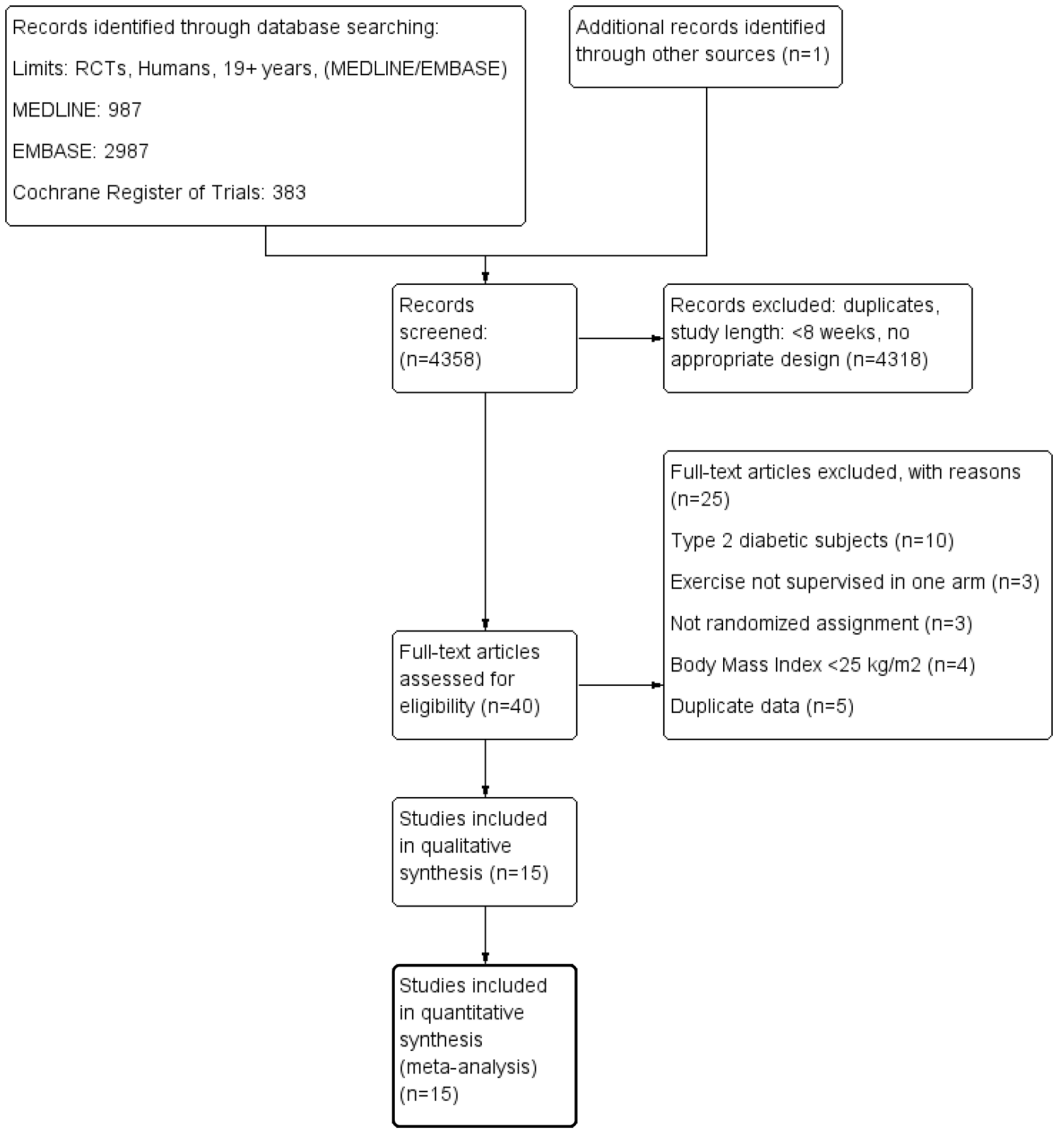
Flow diagram.

Our search strategy and exclusion criteria resulted in a total of 15 trials (17 reports) extracted from 4358 articles that met the objectives and were included in the qualitative and quantitative analysis_ENREF_17_ENREF_17_ENREF_17_ENREF_17_ENREF_17_ENREF_17 [25]–[41]. The detailed steps of the meta-analysis article selection process are described as a flow diagram in Figure 2. Study duration ranging between 2.5 months and 6 months, published between 1994 and 2012 and enrolling a total of 741 participants. The reported mean age varied between 30.5 and 73.2 years, the corresponding BMI values averaged between 27.8 and 33.8 kg/m^2^. Among the 15 trials adopted for meta-analysis, 14 compared RT vs. AET [26]–[41], 4 compared CT vs. AET [25], [26], [32], [33], [38], and 3 compared CT vs. RT [26], [32], [33], [38]. Four studies reported a dietary co-intervention in the AET and RT groups [27], [28], [35], [40]. General study characteristics are summarized in Table 1.

**Table 1:** General study characteristics.

Reference	Sample size,	Mean age (yrs)	Duration (months)	Study design	Exercise prescription	Outcomes (*significant changes between groups) p<0.05
	Mean baseline BMI (kg/m^2^)	Female (%)				
		Male (%)				
Ahmadizad et al. 2007 [30]	16	40.9	3	RT vs.	RT: 11 Ex, 50–60% 1RM, dose: 12 S/MG/W;	RT: ↑ VO_2_ max
	28.1	0%		AET	AET: 75–85% MHR, 60–90 min/wk;	AET: ↑ VO_2_ max
		100%				
Ballor et al. 1996 [39]	18	61	3	RT vs.	RT: 7 Ex, 50–80% 1 RM, 8 R, 9 S/MG/W;	RT: ↑ LBM
	>32	55%		AET	AET: 50% VO_2_ max, 60–180 min/wk;	AET: ↓ BW, FM; ↑ VO_2_ max
		45%				
Banz et al. 2001 [31]	19	47.5	2.5	RT vs.	RT: 8 Ex, 10 R, dose: 9 S/MG/W;	RT: ↓ WHR;
	33	0%		AET	AET: 60–85% MHR, 120 min/wk;	AET: ↓ WHR; ↑ HDL-C
		100%				
Bateman et al. 2011 [32] Willis et al. 2012 [38]	196	49.5	4	RT vs.	RT: 8 Ex, 8–12 R, dose: 9 S/MG/W;	RT: ↑ BW, LBM; ↑ VO_2_ max
	30.5	48%		AET vs.	AET: 65–80% VO_2_ max; 130 min/wk;	AET: ↓ BW, WC, FM, TG; ↑ VO_2_ max
		52%		CT	CT: RT: 8 Ex, 8–12 R, dose: 9 S/MG/W; RT: 65–80% VO_2_ max; 130 min/wk;	CT: ↓ BW, WC, FM, TG; ↑ LBM, VO_2_ max
Davidson et al. 2009 [33]	108	67.7	6	RT vs.	RT: 9 Ex, dose: 3 S/MG/W;	RT: ↓ WC^a^
	30	57%		AET vs.	AET: 60–75% VO_2_ max, 150 min/wk;	AET: ↓BW^a,b^, WC^a,b^, FM^a,b^; ↑ VO_2_ max^a,b^
		43%		CT	CT: RT: 9 Ex, 3 S/MG/W, AET: 60–75% VO_2_ max, 150 min/wk;	CT: ↓BW^a,b^, WC^a^, FM^a,b^; ↑ VO_2_ max^a,b^ *compared with control^a^, RT^b^,
Donges et al. 2010 [34]	76	n.d	2.5	RT vs.	RT: 6 Ex, 75% 1RM, dose: 3 S/MG/W;	RT: ↓ WC ↑ BW, LBM
	27.8	58%		AET	AET: 75% MHR, 150 min/wk;	AET: ↓BW, WC, FM; ↑ LBM
		42%				
Fenkci et al. 2006 [37] Sarsan et al. 2006 [47]	40	42.85	4	RT vs.	RT: 6 Ex, 75–80% 1 RM, 10 R, dose: 9 S/MG/W;	RT: ↓ BW, FM; ↑ LBMAET: ↓ BW, FM
	35	100%		AET	AET: 50–85% HRR; 45–225 min/wk;	AET: ↓ BW, FM
		0%				
Fisher et al. 2011 [40]	97	30.5	until BMI <25 kg/m	RT + CR vs.	RT: 10 Ex, 80% 1 RM, 10 R, dose: 6 S/MG/W;	RT: ↓ BW, WC, TC, TG
	28	100%		AET + CR	AET: 65–80% MHR,150 min/wk;	AET: ↓ BW, WC, TC, TG
		0%				
Janssen et al. 2002 [35]	25	36.15	4	RT + CR vs.	RT: 7 Ex, 8–12 R, dose: 3 S/MG/W;	RT: ↓ BW, WHR, WC, TC, LDL-C, HDL-C
	33.8	100%		AET +CR	AET: 50–85% of MHR, max. 300 min/wk;	AET: ↓ BW, WHR, WC, TC
		0%				
Martins et al. 2010 [36]	32	73.2	4	RT vs.	RT: 8 Ex, 8–15 RM, dose: 3–9 S/MG/W;	RT:/
	30.8	63%		AET	AET: 40–85% HRR, 120 min/wk;	AET: ↓ BW, WC, LDL-C
		37%				
Potteiger et al. 2012 [29]	22	36.4	6	RT vs.	RT: high: 100% 5–7 RM, moderate: 80% 8–10 RM, dose: increase from 3 to max 16 S/MG/W;	RT: ↓ HDL-C, WC, FM; ↑ LBMAET: ↓ BW, WC, TG
	31.2	0%		AET	AET: 65–80%VO_2_max; 135–180 min/wk;	AET: ↓ BW, WC, TG
		100%				
Rice et al. 1999 [28]	20	43.6	4	RT +CR vs.	RT: 7 Ex; 8–12 R, dose: 3 S/MG/W;	RT: ↓ BW, WHR, WC
	33.05	0%		AET +CR	AET: 50–85% of MHR, max 300 min/wk;	AET: ↓ BW, WHR, WC
		100%				
Ross et al. 1994 [27]	24	36.1	4	RT +CR vs.	RT: 8 Ex, 70–80% 1RM, dose: 3 S/MG/W;	RT: ↓ BW, WC
	33.1	100%		AET +CR	AET: 50–85% MHR, 45–180 min/wk;	AET: ↓ BW, WC
		0%				
Stensvold et al. 2010 [26]	32	51.23	3	RT vs.	RT: 15–20 R, dose: 6–9 S/MG/W;	RT: ↓ WC, FM
	31.26	40%		AET vs.	AET: interval training (90–95% VO_2_ max), 130 min/wk;	AET: ↓ WC, FM
		60%		CT	CT: AET (2x wk), RT (1x wk);	CT: ↓ WC; ↑ LBM
Wallace et al. 1997 [25]	16	41.2	3.5	AET vs.	AET: 60–70% HRR, 180 min/wk;	AET: ↑ VO_2_ max,
	94 kg	0%		CT	CT: RT: 8 Ex, 75% 1RM, 8–12 R, dose: 12 S/MG/W; AET: 60–70% HRR, 180 min/wk;	CT: ↓ FM, TG; ↑ HDL-C, VO_2_ max
		100%				

_2_ max, maximal oxygen uptake; WC, waist circumference; WHR, waist to hip ratio; ↑, higher/more; ↓, lower/less. AET, aerobic endurance training; BW, body weight; CR, caloric restriction; CT, combined training (RT and AET); Ex, exercises; FM, fat mass; HDL-C, high-density lipoprotein cholesterol; HRR, heart rate reserve; LBM, lean body mass; LDL-C, low-density lipoprotein cholesterol; MHR, maximum heart rate; n.d, no data; R, Repetition; RT, resistance training; S/MG/W, sets for each muscle group per week; TC, total cholesterol; TG, triacylglycerols; VO

The pooled estimate of effect size for the effects of RT vs. AET, CT vs. AET, and CT vs. RT on anthropometric and cardiovascular risk factor outcomes are summarized in Table 2.

**Table 2:** Pooled estimates of effect size (95% confidence intervals) expressed as weighted mean difference for the effects of AET vs. RT, CT vs. AET and CT vs. RT on anthropometric outcomes, blood lipids and cardiorespiratory fitness.

Outcomes	No. of Studies	Sample Size	MD	95% CI	p-values	Inconsistency I^2^	Egger test
AET vs. RT							
BW (kg)	14	560	−1.15	[−2.23, −0.07]	0.04	34%	0.032
WC (cm)	10	410	−1.10	[−1.85, −0.36]	0.004	0%	0.742
WHR	8	232	−0.01	[−0.02, 0.01]	0.48	82%	0.156
FM (kg)	8	415	−1.14	[−1.83, −0.45]	0.001	3%	0.277
LBM (kg)	7	335	−1.26	[−1.81, −0.71]	<0.00001	0%	0.883
TC (mg/dl)	7	230	−2.40	[−10.29, 5.50]	0.55	0%	0.270
LDL-C (mg/dl)	6	208	−3.69	[−14.91, 7.52]	0.52	46%	0.841
HDL-C (mg/dl)	8	291	1.49	[−0.18, 3.16]	0.08	0%	0.203
TG (mg/dl)	7	272	−7.63	[−22.61, 7.34]	0.32	0%	0.481
VO2max (ml/kg/min)	7	260	2.53	[1.62, 3.44]	<0.00001	0%	0.362
CT vs. AET							
BW (kg)	4	184	0.34	[−0.39, 1.08]	0.36	0%	0.141
WC (cm)	3	168	−0.14	[−1.03, 0.76]	0.77	0%	0.688
FM (kg)	4	184	−0.56	[−1.34, 0.22]	0.16	0%	0.234
LBM (kg)	3	112	0.90	[0.31, 1.48]	0.003	0%	0.600
HDL-C (mg/dl)	3	92	0.76	[−1.30, 2.81]	0.47	0%	0.079
TG (mg/dl)	3	92	0.19	[−19.47, 19.86]	0.98	0%	0.297
VO2max (ml/kg/min)	4	172	−0.04	[−1.47, 1.39]	0.96	25%	0.024
CT vs. RT							
BW (kg)	3	173	−2.03	[−2.94, −1.12]	<0.0001	19%	0.400
WC (cm)	3	173	−1.57	[−2.38, −0.75]	0.0002	0%	0.295
FM (kg)	3	173	−1.88	[−2.67, −1.08]	<0.00001	9%	0.297
VO2max (ml/kg/min)	3	162	2.79	[1.78, 3.79]	<0.00001	0%	0.102

_2_ max, maximal oxygen uptake; WC, waist circumference; WHR, waist to hip ratio. BW, body weight; CRP; FM, fat mass; HDL-C, high density lipoprotein cholesterol; LBM, lean body mass; LDL-C, low density lipoprotein cholesterol; TC, total cholesterol; TG, triacyglycerols; VO

### 

#### Pairwise meta-analysisAET vs. RT

The reduction of BW [MD: -1.15 kg (95% CI −2.23 to −0.07), p = 0.04] (I^2^ = 34%) (Figure S1 in File S1), WC [MD: -1.10 cm (95% CI −1.85 to −0.36), p = 0.004] (I^2^ = 0%) (Figure S2 in File S1), and FM [MD: -1.14 kg (95% CI −1.83 to −0.45), p = 0.001] (I^2^ = 3%) (Figure S3 in File S1) was significantly more pronounced in the AET groups as compared to RT, respectively. However, participants in the RT groups showed a significantly more distinct increase in LBM [MD: 1.26 kg (95% CI 0.71 to 1.81), p<0.00001] (I^2^ = 0%), when compared to AET protocols (Figure S4 in File S1). Comparison of AET with RT did not result in significantly different outcomes with respect to WHR.

#### CT vs. AET

CT significantly increased LBM [MD: 0.90 kg (95% CI 0.31 to 1.48), p = 0.003] (I^2^ = 0%) (Figure S5 in File S1) when compared to the corresponding effects of AET. No other anthropometric parameter was affected in a different fashion by either CT or AET.

#### CT vs. RT

CT protocols were associated with a significantly more substantial reduction in BW [MD: -2.03 kg (95% CI −2.94 to −1.12), p<0.0001] (I^2^ = 19%) (Figure S6 in File S1), WC [MD: -1.57 cm (95% CI −2.38 to −0.75), p = 0.0002] (I^2^ = 0%) (Figure S7 in File S1), and FM [MD: -1.88 kg (95% CI −2.67 to −1.08), p<0.00001] (I^2^ = 9%) (Figure S8 in File S1) when compared to RT strategies, respectively. No significant differences were observed with regard to LBM.

#### Blood lipids and cardiorespiratory fitness

VO_2_max as an indicator of cardiorespiratory fitness was significantly more improved following AET [MD: 2.53 ml/kg/min (95% CI 1.62 to 3.44), p<0.00001] (I^2^ = 0%) (Figure S9 in File S1) and CT procedures [MD: 2.79 ml/kg/min (95% CI 1.78 to 3.79), p<0.00001] (I^2^ = 0%) (Figure S10 in File S1) when compared to RT interventions, respectively. No significant differences were observed for TC, LDL-C, HDL-C, and TG, respectively (Table 2).

### Network meta-analysis

The pooled estimates of effect size for the comparison of AET vs. RT vs. CT using both direct and indirect evidence on anthropometric and cardiovascular risk factor outcomes are summarized in Table 3 (except LDL-C, since only AET vs. RT trials were available). For each outcome, a common between-study heterogeneity parameter was assumed, to reflect the variability between studies of all interventions (Table 3). The ranking probabilities, and rankings with credibility intervals of AET, RT, and CT for each outcome is presented in Table 4.

**Table 3:** Mean differences estimated from the random effects network meta-analysis model

	BW (kg)	WC (cm)	WHR	FM (kg)	LBM (kg)
AET versus					
RT	−1.34 [−2.28, 0.094]	−1.3[−2.45, 0.058]	−0.006 [−0.022, 0.011]	−1.00[−1.90, 0.34]	−1.30 [−3.24, 0.74]
CT versus					
AET	−0.22 [−2.21, 1.11]	−0.22[−2.09, 1.29]	−0.049 [−0.10, 0.009]	−0.72[−2.20, 0.469]	0.75 [−2.99, 2.77]
CT versus					
RT	−1.59 [−3.17, 0.058]	−1.54 [−3.32, 0.015]	−0.056 [−0.11, 0.006]	−1.73[−2.92, −0.30]	−0.53 [−4.36, 1.59]
I^2^	0.817 [0.04, 2.44]	0.72 [0.038, 2.66]	0.016 [0.0082, 0.04]	0.49 [0.025, 2.26]	0.86 [0.041, 4.55]
					
	TC (mg/dl)	HDL-C (mg/dl)	TG (mg/dl)	VO2 max (ml/kg/min)	
AET versus					
RT	−3.82 [−15.49, 6.66]	1.44 [−0.60, 3.38]	−10.8 [−30.22, 8.12]	2.67 [1.47, 3.97]	
CT versus					
AET	10.72[−15.38, 36.84]	0.86 [−1.64, 3.62]	0.22 [−24.22, 28.98]	−0.019 [−1.74, 1.28]	
CT versus					
RT	6.88 [−20.43, 33.24]	2.30 [−0.54, 5.29]	−10.56 [−37.51, 20.39]	2.66 [1.00, 3.99]	
I^2^	5.87 [0.299, 23.88]	0.78 [0.046, 3.26]	9.79 [0.38, 36.97]	0.519 [0.023, 2.24]	

% credible intervals); I^2^: estimated between study heterogeneity standard deviation (95% credible intervals); Relative intervention effectiveness is expressed as posterior medians (95

AET, aerobic exercise training; BW, body weight; CT, combined training; FM, fat mass; HDL-C, high density lipoprotein cholesterol; LBM, lean body mass; RT, resistance training; TC, total cholesterol; TG, triacyglycerols; VO2 max, maximal oxygen uptake; WC, waist circumference; WHR, waist to hip ratio;

**Table 4:** Ranking probabilities of AET, RT, and CT for the different outcome parameters.

	Rank		Probabilities		
	median	95% CrI	best	2^nd^ best	worst
**BW**					
AET	2	[1], [3]	35.9%	61.4%	2.7%
RT	3	[1], [3]	0.6%	4.7%	94.7%
CT	1	[1], [3]	63.5%	34%	2.5%
**WC**					
AET	2	[1], [3]	35.8%	62.3%	1.9%
RT	3	[1], [3]	0.4%	3.8%	95.7%
CT	1	[1], [3]	63.7%	33.9%	2.4%
**WHR**					
AET	2	[1], [3]	3.5%	77.2%	19.2%
RT	3	[1], [3]	1.8%	20.7%	77.5%
CT	1	[1], [3]	94.7%	2.1%	3.2%
**FM**					
AET	2	[1], [3]	8.7%	86%	5.2%
RT	3	[1], [3]	0.7%	5.5%	93.8%
CT	1	[1], [3]	90.5%	8.5%	1%
**LBM**					
AET	3	[1], [3]	4.3%	20%	75.6%
RT	1	[1], [3]	74.3%	22.5%	3.2%
CT	2	[1], [3]	21.3%	57.4%	21.2%
**TC**					
AET	1	[1], [3]	64%	30.5%	5.5%
RT	2	[1], [3]	18.1%	56.2%	25.7%
CT	3	[1], [3]	17.9%	13.3%	68.8%
**HDL-C**					
AET	2	[1], [3]	22.6%	70.8%	6.6%
RT	3	[1], [3]	1.9%	9.1%	89%
CT	1	[1], [3]	75.5%	20.1%	4.4%
**TG**					
AET	2	[1], [3]	45.9%	46.6%	7.4%
RT	3	[1], [3]	6.4%	21.2%	72.4%
CT	2	[1], [3]	47.6%	32.2%	20.2%
**VO_2_max**					
AET	1	[1], [3]	51.1%	48.9%	0%
RT	3	[1], [3]	0%	0.5%	99.5%
CT	2	[1], [3]	48.9%	50.6%	0.5%

AET, aerobic exercise training; BW, body weight; CrI, credible intervals; CT, combined training; FM, fat mass; HDL-C, high density lipoprotein cholesterol; LBM, lean body mass; RT, resistance training; TC, total cholesterol; TG, triacyglycerols; VO2 max, maximal oxygen uptake; WC, waist circumference; WHR, waist to hip ratio;

Both AET and CT were more effective in reducing body weight compared to RT (Figure S11 in File S1). The network meta-analysis showed that CT was the most powerful exercise intervention to reduce WC (Figure S12 in File S1), and FM (Figure S13 in File S1). Regarding LBM, the observations of the pairwise meta-analysis were also confirmed. No significant differences were observed for WHR, and blood lipids. Maximal oxygen uptake was significantly more pronounced in the AET and CT groups as compared to the RT groups (Figure S14 in File S1).

Due to the structure of the evidence, inconsistency between direct and indirect evidence was only possible for the BW outcome. No evidence of inconsistency was found with Bayesian p-values for the difference between direct and indirect evidence all greater than 0.90.

### Sensitivity analysis

Sensitivity analysis were performed for obesity, age (≥50 years vs. <50 years) and gender. The primary analysis was confirmed when including only obese subjects. Inclusion of older people (≥50 years) resulted in slightly more pronounced effects compared to younger (<50 years), while no gender specific differences were observed (data not shown). Furthermore, a meta-analysis of change scores was performed for those trials reporting the corresponding data (10 of 15, see Table S1 in File S1).

### Study quality

Three studies were excluded, since study participants were not assigned to intervention groups via randomization [42], [43]. Except for one study [34] (semi-randomization) all others were randomized, but only 4 studies reported random sequence generation and only 1 trial reported allocation concealment. None of the studies reported blinding of participants, a lack of which is a common characteristic in exercise interventions (Figure 1). Only one trial performed intention to treat analysis, and appears to have adequate blinding outcome assessment [33]. The retention rate ranged from 63% to 92%, with no significant differences between exercise groups.

### Publication Bias

The Begg's and Egger's linear regression tests provided evidence for a potential publication bias for BW (p = 0.032) following comparison of AET vs. RT, and for VO_2_ max following comparison of CT vs. AET (p = 0.024). Funnel plots were generated for outcome measures provided by at least 10 different trials (see Figures S15-S16 in File S1). The plots (with respect to effect size changes for outcome parameters BW (Figure S15 in File S1), and WC (Figure S16 in File S1) in response to training modalities AET vs. RT indicate moderate asymmetry, suggesting that publication bias cannot be completely excluded as a factor of influence on the present meta-analysis. It remains possible that small studies with inconclusive results have not been published or failed to do so.

## Discussion

To our knowledge, this is the first systematic review investigating the pooled effects of different exercise interventions on anthropometric outcomes, blood lipids and cardiorespiratory fitness. The main findings of this meta-analysis suggest that in subjects with a BMI ≥25 kg/m^2^, AET is more efficient in reducing BW, WC and FM as well as in increasing VO_2_max uptake when compared to RT, respectively. However, RT turned out to be more suitable when it comes to an improvement of lean body mass. Furthermore, the present results provide evidence that a combined intervention seems to be the most promising tool for management of overweight and obesity. CT was more powerful in reducing anthropometric risk factors like BW, WC or FM when compared to RT, and more effective in raising LBM when compared to AET. Pooled direct and indirect evidence on these three exercise interventions showed that CT was the most efficacious to reduce anthropometric outcomes such BW, WC and FM (with the respective ranking probabilities, following Bayesian network meta-analysis: 63%, 63% and 90%).

Since waist circumference correlates with abdominal fat mass and is considered to be an independent predictor of CDV, it can be used as a surrogate marker of abdominal fat mass [44], [45]._ENREF_52 De Koning et al. reported a 2%-increase in CVD risk for each 1cm-gain in WC [45]. By transferring these findings to the results of the present meta-analyses, AET was associated with a reduction in CVD risk that was approximately 2% stronger as in the respective RT counterparts. Moreover, CT resulted in a decline in CVD risk that was by 4% more distinct as compared to the effects of an RT intervention. Aerobic exercise is known to increase the sympathetic tone, and the subsequent release of adrenergic transmitters leads to an increased lipolysis especially in abdominal fat [46].

Regarding lean body mass, the results of the present meta-analyses show that both RT and CT are more effective in raising LBM when compared to AET, respectively. An increase in LBM contributes to the maintenance or may even reflect an increase in resting metabolic rate [47]. Apparently, RT triggers the preservation and buildup of body protein thereby altering the relationship between LBM and FM [48]. In a previous study, it was shown that, if performed twice a week, RT facilitated an increase in LBM by 1−2 kg in the course of 6 months and could prevent age-associated loss of LBM [49].

Results suggest that exercise interventions containing aerobic sessions (whether isolated or as part of a combination training) improve cardiorespiratory fitness when compared to RT as a single training modality. A gain in cardiorespiratory fitness is known to be associated with reduced cardiovascular mortality and cancer incidence in men and women [50], [51]. A pooled analysis by Kodama et al. [52] _ENREF_66 investigating the impact of cardiorespiratory fitness on all-cause mortality and cardiovascular events revealed that an increase in VO_2_ max in the amount of one metabolic equivalent correlated with a 13%-reduction of all-cause mortality as well as with a 15%-decrease in CHD/CVD risk, respectively. The authors suggested that the +1-MET-improving effects on VO_2_ max are comparable to corresponding influences of a decrease in WC (-7 cm), SBP (-5 mmHg), TG (-88 mg/dl), and FG (−18 mg/dl) as well as to increases in HDL-C (+7.72 mg/dl), respectively. When applying these findings to the results of the present meta-analysis, AET outperformed RT as a single training modality with a further 7.5%-risk reduction in all-cause mortality and a further 8.5%-risk reduction in CHD/CVD, respectively.

The present systematic review has several strengths and weaknesses. The meta-analysis were conducted following a stringent protocol, i.e. in all trials, participants were randomly assigned to the intervention groups, and only supervised training protocols were included. Randomized controlled trials are considered to be the gold standard for evaluating the effects of an intervention and are subject to fewer biases as compared to observational studies. The network meta-analysis included all individuals for each outcome. Moreover the present meta-analysis had a substantial sample size (range: 323 to 664) volunteers, thus providing the power to detect statistically significant mean differences as well as to assess publication bias. Network meta-analysis methods were used to obtain coherent estimates of all treatments relative to each other, using all available evidence and adequately accounting for evidence from 3-arm trials (i.e. avoiding the repeated use of data from such trials in different comparisons). This is of particular importance in this application where there were several trials simultaneously comparing all the interventions. Overall, the estimated between-studies heterogeneity parameters were small for all networks, and there was no evidence of inconsistency, which further strengthens the conclusions. Trial characteristics suggest the consistency/similarity assumption is satisfied, which is confirmed by the statistical analysis.

Limitations of the present review include the limited number of studies and the heterogeneity of the study designs. The trials covered in the meta-analyses showed variations in population characteristics (e.g. overweight, obese, age, number and ratio of male and female participants).

A considerable confounder could be the volume of exercise (min/week) prescribed. Two studies reported exercise intensity in the CT group to be twice as high as compared to their respective RT and/or AET counterparts [25], [32], [38]. However, a sensitivity analysis excluding these studies confirmed the results of the primary analysis. Other potential confounders included differences in dietary intake and activity performed outside the monitoring and supervision by the investigators. Most studies reported the method of randomization as well as other data required for risk of bias assessment, which might be due to the fact that the trials were performed within the previous 20 years (between 1994 and 2012). However, another major limitation is the size of the study population, i.e. 11 of the 15 trials had a sample size of less than 60 participants, demanding a conservative interpretation of the results. With respect to publication bias, funnel plots for this systematic review showed low to moderate asymmetry suggesting that e.g. lack of published trials with inconclusive results cannot be completely excluded as a confounder of the present meta-analysis (Figure S15-16 in File S1). According to the results of the Begg's and Egger's linear regression tests, there is evidence for a potential publication bias for BW following pairwise comparison of AET vs. RT and VO_2_ max following direct comparison of CT vs. AET. Therefore, these results should be interpreted with caution. Future trials should focus on high-quality methodological assessment (allocation concealment, blinding of outcome assessment, and intention-to-treat analysis), long-term effects (≥12 months), and larger sample size.

In conclusion, the present systematic review and meta-analysis focused on RCTs mutually comparing AET, RT, and CT. Anthropometrical as well as cardiorespiratory fitness parameters turned out to be significantly more improved following AET or CT protocols as compared to their respective RT counterparts. With respect to the limitations of the present systematic review, a conservative interpretation of the data is required. The primary objective in obesity management is the reduction of body fat. According to the results of the pairwise meta-analysis, reduction of fat mass was significantly more pronounced following AET, and CT as compared to RT. However, addition of RT to AET strategies may prevent loss of LBM, which is a common problem in the course of weight loss in obesity management programs. Evidence from the network meta-analysis suggests that CT is the most efficacious exercise modality in the prevention and treatment of overweight, and obesity and should therefore recommended whenever possible.

## Supporting Information

File S1 **Figure S1: Forest plot showing pooled MD with 95% CI for body weight (kg) for 14 randomized controlled aerobic exercise (AET) vs. resistance (RT) groups.** Figure S2: Forest plot showing pooled MD with 95% CI for waist circumference (cm) for 10 randomized controlled aerobic exercise (AET) vs. resistance (RT) groups. Figure S3. Forest plot showing pooled MD with 95% CI for fat mass (kg) for 8 randomized controlled aerobic training vs. resistance training trials. Figure S4: Forest plot showing pooled MD with 95% CI for lean body mass (kg) for 7 randomized controlled aerobic exercise (AET) vs. resistance (RT) groups. Figure S5. Forest plot showing pooled MD with 95% CI for lean body mass (kg) for 3 randomized controlled combined training vs. aerobic training trials. Figure S6. Forest plot showing pooled MD with 95% CI for body weight (kg) for 3 randomized controlled combined training vs. resistance training trials. Figure S7. Forest plot showing pooled MD with 95% CI for waist circumference (cm) for 3 randomized controlled combined training vs. resistance training trials. Figure S8. Forest plot showing pooled MD with 95% CI for fat mass (kg) for 3 randomized controlled combined training vs. resistance training trials. Figure S9.Forest plot showing pooled MD with 95% CI for maximal oxygen uptake (ml//kg/min) for 7 randomized controlled aerobic exercise (AET) vs. resistance (RT) groups. Figure S10. Forest plot showing pooled MD with 95% CI for maximal oxygen uptake (ml/min/kg) for 3 randomized controlled combined training vs. resistance training trials. Figure S11. Scatterplot showing the median with 95% CrI for body weight (kg) for the indirect comparison of different training modalities. Figure S12. Scatterplot showing the median with 95% CrI for waist circumference (cm) for the indirect comparison of different training modalities. Figure S13. Scatterplot showing the median with 95% CrI for fat mass (kg) for the indirect comparison of different training modalities. Figure S14. Scatterplot showing the median with 95% CrI for maximal oxygen uptake (ml/kg/min) for the indirect comparison of different training modalities. Figure S15. Funnel plot showing study precision against the MD effect estimate with 95% CIs for body weight (aerobic vs. resistance training). Figure S16. Funnel plot showing study precision against the MD effect estimate with 95% CIs for waist circumference (aerobic vs. resistance training). Table S1. Pooled estimates (change scores) of effect size (95% confidence intervals) expressed as weighted mean difference for the effects of AET vs. RT, CT vs. AET and CT vs. RT on anthropometric outcomes, blood lipids and cardiorespiratory fitness. Table S2. Baseline study comparability reported as mean and standard deviation.
(DOCX)
