# Supplementary material for: Impact of Different Training Modalities on Anthropometric and Metabolic Characteristics in Overweight/Obese Subjects: A Systematic Review and Network Meta-Analysis
Source: PLoS One. 2013 Dec 17;8(12):e82853. doi: 10.1371/journal.pone.0082853 (PMC3866267; doi:10.1371/journal.pone.0082853)
Supplement: File S1 — Figure S1: Forest plot showing pooled MD with 95% CI for body weight (kg) for 14 randomized controlled aerobic exercise (AET) vs. resistance (RT) groups. Figure S2: Forest plot showing pooled MD with 95% CI for waist circumference (cm) for 10 randomized controlled aerobic exercise (AET) vs. resistance (RT) groups. Figure S3. Forest plot showing pooled MD with 95% CI for fat mass (kg) for 8 randomized controlled aerobic training vs. resistance training trials. Figure S4: Forest plot showing pooled MD with 95% CI for lean body mass (kg) for 7 randomized controlled aerobic exercise (AET) vs. resistance (RT) groups. Figure S5. Forest plot showing pooled MD with 95% CI for lean body mass (kg) for 3 randomized controlled combined training vs. aerobic training trials. Figure S6. Forest plot showing pooled MD with 95% CI for body weight (kg) for 3 randomized controlled combined training vs. resistance training trials. Figure S7. Forest plot showing pooled MD with 95% CI for waist circumference (cm) for 3 randomized controlled combined training vs. resistance training trials. Figure S8. Forest plot showing pooled MD with 95% CI for fat mass (kg) for 3 randomized controlled combined training vs. resistance training trials. Figure S9.Forest plot showing pooled MD with 95% CI for maximal oxygen uptake (ml//kg/min) for 7 randomized controlled aerobic exercise (AET) vs. resistance (RT) groups. Figure S10. Forest plot showing pooled MD with 95% CI for maximal oxygen uptake (ml/min/kg) for 3 randomized controlled combined training vs. resistance training trials. Figure S11. Scatterplot showing the median with 95% CrI for body weight (kg) for the indirect comparison of different training modalities. Figure S12. Scatterplot showing the median with 95% CrI for waist circumference (cm) for the indirect comparison of different training modalities. Figure S13. Scatterplot showing the median with 95% CrI for fat mass (kg) for the indirect comparison of different training modalities. Figur [file pone.0082853.s001.docx]

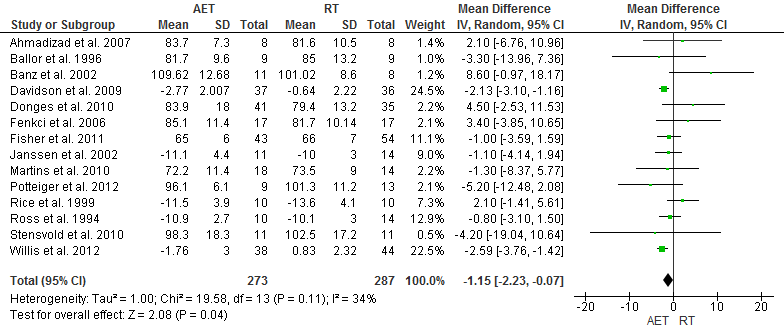
Figure S1: Forest plot showing pooled MD with 95% CI for body weight (kg) for 14 randomized controlled aerobic exercise (AET) vs. resistance (RT) groups.

For each AET study, the shaded square represents the point estimate of the intervention effect. The horizontal line joins the lower and upper limits of the 95% CI of these effects. The area of the shaded square reflects the relative weight of the study in the respective meta-analysis. The diamond at the bottom of the graph represents the pooled MD with the 95% CI for the 14 study groups.

Figure S2: Forest plot showing pooled MD with 95% CI for waist circumference (cm) for 10 randomized controlled aerobic exercise (AET) vs. resistance (RT) groups.


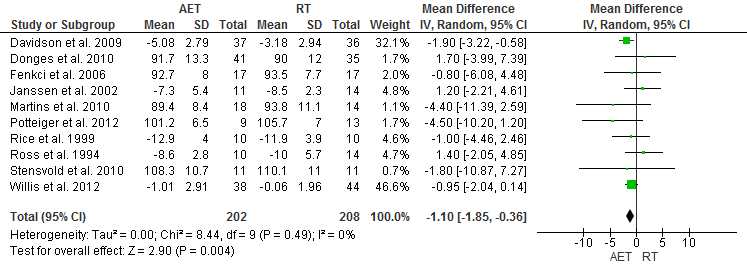


Figure S3. Forest plot showing pooled MD with 95% CI for fat mass (kg) for 8 randomized controlled aerobic training vs. resistance training trials.


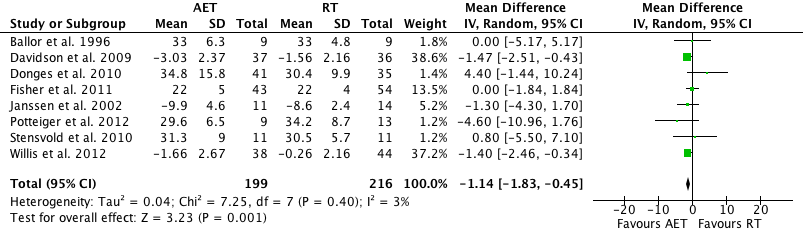


Figure S4: Forest plot showing pooled MD with 95% CI for lean body mass (kg) for 7 randomized controlled aerobic exercise (AET) vs. resistance (RT) groups.


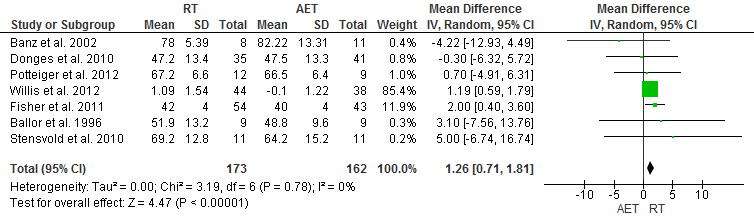


Figure S5. Forest plot showing pooled MD with 95% CI for lean body mass (kg) for 3 randomized controlled combined training vs. aerobic training trials.


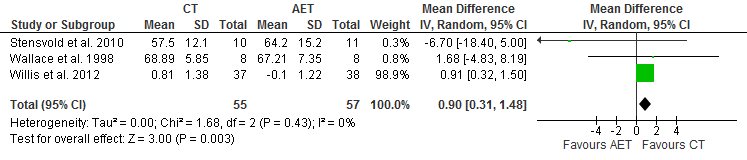


Figure S6. Forest plot showing pooled MD with 95% CI for body weight (kg) for 3 randomized controlled combined training vs. resistance training trials.


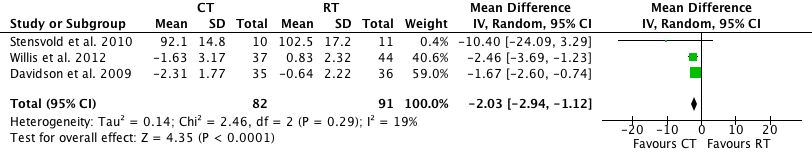


Figure S7. Forest plot showing pooled MD with 95% CI for waist circumference (cm) for 3 randomized controlled combined training vs. resistance training trials.


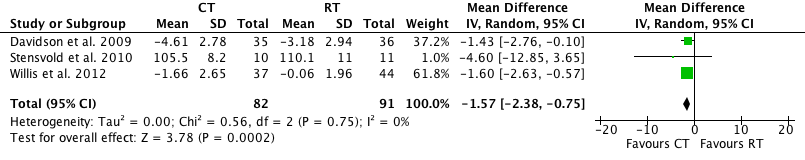


Figure S8. Forest plot showing pooled MD with 95% CI for fat mass (kg) for 3 randomized controlled combined training vs. resistance training trials.


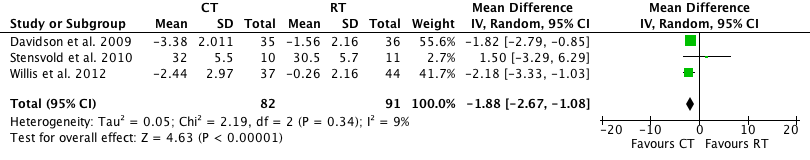


Figure S9.Forest plot showing pooled MD with 95% CI for maximal oxygen uptake (ml//kg/min) for 7 randomized controlled aerobic exercise (AET) vs. resistance (RT) groups.


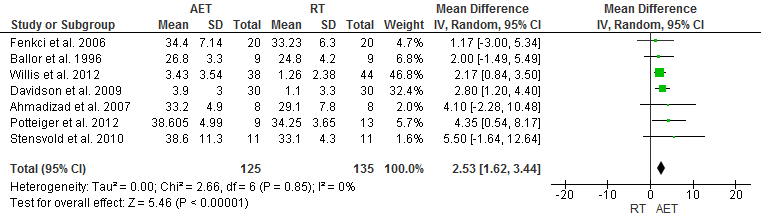


Figure S10. Forest plot showing pooled MD with 95% CI for maximal oxygen uptake (ml/min/kg) for 3 randomized controlled combined training vs. resistance training trials.


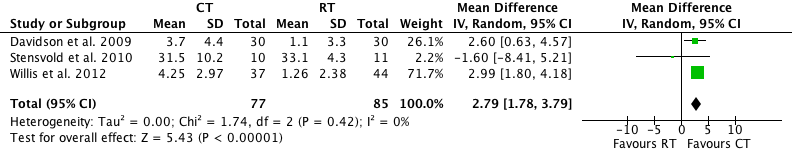


Figure S11. Scatterplot showing the median with 95% CrI for body weight (kg) for the indirect comparison of different training modalities.

AET, aerobic exercise; CrI, credible intervals; CT, combined training; RT; resistance training;

Figure S12. Scatterplot showing the median with 95% CrI for waist circumference (cm) for the indirect comparison of different training modalities.

AET, aerobic exercise; CrI, credible intervals; CT, combined training; RT; resistance training;

Figure S13. Scatterplot showing the median with 95% CrI for fat mass (kg) for the indirect comparison of different training modalities.

AET, aerobic exercise; CrI, credible intervals; CT, combined training; RT; resistance training;

Figure S14. Scatterplot showing the median with 95% CrI for maximal oxygen uptake (ml/kg/min) for the indirect comparison of different training modalities.

AET, aerobic exercise; CrI, credible intervals; CT, combined training; RT; resistance training;

Figure S15. Funnel plot showing study precision against the MD effect estimate with 95% CIs for body weight (aerobic vs. resistance training). SE = Standard error

Figure S16. Funnel plot showing study precision against the MD effect estimate with 95% CIs for waist circumference (aerobic vs. resistance training). SE = Standard error

| **Outcomes** | **No. of Studies** | **Sample Size** | **WMD** | **95% CI** | **p-values** | **Inconsistency I^2^** |
| --- | --- | --- | --- | --- | --- | --- |
| **AET vs. RT** | | | | | | |
| BW (kg) | 9 | 380 | -1.64 | [-2.27, -1.01] | <0.00001 | 30% |
| WC (cm) | 8 | 362 | -0.92 | [-1.80, -0.05] | 0.04 | 40% |
| WHR | 6 | 302 | 0.00 | [-0.00, 0.00 | 0.96 | 0% |
| FM (kg) | 6 | 296 | -0.95 | [-1.49, -0.42] | 0.0005 | 0% |
| LBM (kg) | 4 | 198 | -1.05 | [-1.56, -0.53] | <0.0001 | 16% |
| TC (mg/dl) | 3 | 123 | -8.02 | [-21.82, 5.78] | 0.25 | 65% |
| HDL-C (mg/dl) | 4 | 184 | 1.36 | [-0.26, 2.98] | 0.10 | 0% |
| TG (mg/dl) | 4 | 184 | -15.07 | [-32.23, 2.10] | 0.09 | 0% |
| VO2max (ml/kg/min) | 5 | 522 | 2.23 | [1.35, 3.11] | <0.00001 | 0% |
| **CT vs. AET** | | | | | | |
| BW (kg) | 4 | 184 | 0.53 | [-0.16, 1.23] | 0.13 | 0% |
| WC (cm) | 3 | 168 | 0.13 | [-0.64, 0.89] | 0.75 | 9% |
| FM (kg) | 4 | 184 | -0.12 | [-1.42, 1.18] | 0.86 | 66% |
| LBM (kg) | 3 | 122 | 0.88 | [0.35, 1.41] | 0.001 | 0% |
| HDL-C (mg/dl) | 3 | 92 | 1.13 | [-1.13, 3.39] | 0.33 | 0% |
| TG (mg/dl) | 3 | 92 | -6.51 | [-27.43, 14.41] | 0.54 | 0% |
| VO2max (ml/kg/min) | 4 | 172 | 0.28 | [-0.76, 1.32] | 0.60 | 0% |
| **CT vs. RT** | | | | | | |
| BW (kg) | 3 | 173 | -1.24 | [-2.83, 0.35] | 0.13 | 77% |
| WC (cm) | 3 | 173 | -0.77 | [-2.27, 0.72] | 0.31 | 81% |
| FM (kg) | 3 | 173 | -0.91 | [-2.98, 1.15] | 0.38 | 91% |
| VO2max (ml/kg/min) | 3 | 162 | 2.79 | [1.82, 3.76] | <0.00001 | 0% |

Table S1: Pooled estimates (change scores) of effect size (95% confidence intervals) expressed as weighted mean difference for the effects of AET vs. RT, CT vs. AET and CT vs. RT on anthropometric outcomes, blood lipids and cardiorespiratory fitness.

BW, body weight; FM, fat mass; HDL-C, high density lipoprotein cholesterol; LBM, lean body mass; LDL-C, low density lipoprotein cholesterol; TC, total cholesterol; TG, triacyglycerols; VO_2_ max, maximal oxygen uptake; WC, waist circumference; WHR, waist to hip ratio.

| **Reference**  Table S2. Baseline study comparability reported as mean and standard deviation. | **BW**  **(kg)** | **WC**  **(cm)** | **WHR** | **FM**  **(kg)** | **LBM**  **(kg)** | **TC**  **(mg/dl)** | **LDL-C**  **(mg/dl)** | **HDL-C**  **(mg/dl)** | **TG**  **(mg/dl)** | **VO_2_max**  **(ml/kg/min)** |
| --- | --- | --- | --- | --- | --- | --- | --- | --- | --- | --- |
| Ahmadizad et al. 2007 [[27](#_ENREF_27)]  No significant differences between groups for any baseline variable | AET: 83.1±6.8  RT: 82.3±10 | n.d  n.d | AET: 0.95±0.006  RT: 0.95±0.004 | n.d  n.d | n.d  n.d | n.d  n.d | n.d  n.d | n.d  n.d | n.d  n.d | AET: 26.3±4.8  RT: 24.6±8.8 |
| Ballor et al. 1996 [[45](#_ENREF_45)]  No significant differences between groups for any baseline variable | AET: 84.2±9.9  RT: 84.6±12 | n.d  n.d | AET: 0.86±0.09  RT: 0.87±0.09 | AET: 34.8±5.7  RT: 34.2±4.8 | AET: 49.4±9.9  RT: 50.4±13.2 | n.d  n.d | n.d  n.d | n.d  n.d | n.d  n.d | AET: 23.9±4.8  RT: 23.4±3.6 |
| Banz et al. 2001 [[30](#_ENREF_30)]  No significant differences between groups for any baseline variable | AET: 109.35±12.15  RT: 101.25±9.45 | n.d  n.d | AET: 1.01±0.04  RT: 1.03±0.03 | n.d  n.d | AET: 81.4±11.56  RT: 74.92±5.58 | AET: 205±44.3  RT: 203±41.6 | AET: 129.8±56.7  RT: 93±70.4 | AET: 29.8±7  RT: 31.7±8.4 | n.d  n.d | n.d  n.d |
| Bateman et al. 2011 [[31](#_ENREF_31)]  Willis et al. 2012 [[44](#_ENREF_44)]  No significant differences between groups for any baseline variable | AET: 89.3±10.8  RT: 89.2±14.5  CT: 90.1±13.2 | AET: 96.1±10.25  RT: 93.6±9.06  CT: 97.3±8.9 | n.d  n.d  n.d | AET: 34.7±7.89  RT: 34.3±9.12  CT: 34.9±8.92 | AET: 53.3±8.71  RT: 54.4±13.3  CT: 54±9.59 | n.d  n.d  n.d | n.d  n.d  n.d | AET: 41.5±14.2  RT: 46.8±13.9  CT: 45±11 | AET: 154±81.3  RT: 140±81  CT: 152±93.9 | AET: 27.3±5.57  RT: 27±6.24  CT: 27±5.78 |
| Davidson et al. 2009 [[35](#_ENREF_35)]  Female vs. male  No significant differences between groups for any baseline variable | n.d  n.d  n.d | AET: 104.2±10.4  RT: 104.3±8.5  CT: 102.8±9.6  AET: 113±7.9  RT: 111±5.4  CT: 114.1±8.3 | n.d  n.d  n.d | AET: 34.9±7.2  RT: 36.4±6.7  CT: 34.9±6.8  AET: 33±9.2  RT: 31.5±4.5  CT: 34.9±6.8 | n.d  n.d  n.d | n.d  n.d  n.d | n.d  n.d  n.d | n.d  n.d  n.d | n.d  n.d  n.d | AET: 21.8±3.1  RT: 21±3.9  CT: 23.5±5.2  AET: 26.9±4  RT: 27.8±5.2  CT: 28.1±6.2 |
| Donges et al.  2010 [[36](#_ENREF_36)]  No significant differences between groups for any baseline variable | AET: 84.8±18.6  RT: 78.6±12.9 | AET: 94±14.7  RT: 91.3±11.9 | AET: 0.86±0.09  RT: 0.87±0.09 | AET: 36.1±15.1  RT: 31.3±9.4 | AET: 46.8±13.4  RT: 45.5±12.0 | AET: 205.4±35.1  RT: 199.2±37.83 | AET: 128.57±27.41  RT: 123.16±28.95 | AET: 51.73±12.35  RT: 54.05±17.76 | AET: 121.34±71.74  RT: 108.94±64.65 | n.d  n.d |
| Fenkci et al. 2006 [[42](#_ENREF_42)]  Sarsan et al. [47]  No significant differences between groups for any baseline variable | AET: 88.2±12.6  RT: 85.4±9.92 | AET: 94.6±9.1  RT: 96.2±8.1 | AET: 0.83±0.1  RT: 0.87±0.1 | n.d  n.d | n.d  n.d | AET: 207.9±30.8  RT: 213.7±35.5 | AET: 139.4±43  RT: 131.1±33.6 | AET: 51±13.6  RT: 53.4±12.3 | AET: 124.1±38.2  RT: 118±56.1 | AET: 27.09±5.13  RT: 27.19±5.59 |
| Fisher et al. 2011  [[46](#_ENREF_46)]  No significant differences between groups for any baseline variable | AET: 77±7  RT: 78±8 | n.d  n.d | n.d  n.d | AET: 34±5  RT: 33±5 | AET: 40±4  RT: 41±4 | n.d  n.d | n.d  n.d | n.d  n.d | n.d  n.d | n.d  n.d |
| Janssen et al. 2002 [[37](#_ENREF_37)]  No significant differences between groups for any baseline variable | AET: 99.9±19.9  RT: 86.1±10.5 | AET: 101.9±13.9  RT: 95.6±9 | AET: 0.81±0.06  RT: 0.81±0.04 | AET: 47.3±15.7  RT: 37.8±9.3 | n.d  n.d | AET: 178.76±33.97  RT: 190.73±30.11 | AET: 110.42±27.41  RT: 119.69±30.11 | AET: 41.69±10.03  RT: 44.78±10.03 | AET: 132.86±67.31  RT: 128.43±68.2 | n.d  n.d |
| Martins et al. [[40](#_ENREF_40)]  No significant differences between groups for any baseline variable | AET: 73.1±11.9  RT: 73.7±9.2 | AET: 93.3±9.9  RT: 94.6±10.4 | n.d  n.d | n.d  n.d | n.d  n.d | AET: 208.3±36.3  RT: 218.5±33.6 | AET: 79.8±32  RT: 90.9±27.5 | AET: 51.8±9.4  RT: 52.5±9.7 | n.d  n.d | RT vs. AET |
| Potteiger et al. 2012 [[25](#_ENREF_25)]  No significant differences between groups for any baseline variable | AET: 102±9.1  RT: 101.3±11.4 | AET: 106.8±7.3  RT: 108.4±9 | n.d  n.d | AET: 35.2±8.3  RT: 36.2±8.8 | AET: 66.8±5.7  RT: 65.6±6.7 | AET: 185.71±39.38  RT: 198.45±41.31 | AET: 111.58±23.55  RT: 126.64±30.88 | AET: 42.85±9.26  RT: 39.38±5.4 | AET: 170.94±79.71  RT: 136.4±62 | AET: 32.64±4.21  RT: 34.45±5.82 |
| Rice et al. 1999 [[24](#_ENREF_24)]  No significant differences between groups for any baseline variable | AET: 100.9±12.7  RT: 109.9±9.2 | AET: 113.4±7.7  RT: 118.5±10.7 | AET: 1.01±0.06  RT: 1.02±0.04 | n.d  n.d | n.d  n.d | n.d  n.d | n.d  n.d | n.d  n.d | n.d  n.d | n.d  n.d |
| Ross et al. 1994 [[23](#_ENREF_23)]  No significant differences between groups for any baseline variable | AET: 96±16.3  RT: 86.1±10.5 | AET: 110.6±5.1  RT: 112.4±10.6 | AET: 0.91±0.07  RT: 0.95±0.06 | n.d  n.d | n.d  n.d | n.d  n.d | n.d  n.d | n.d  n.d | n.d  n.d | n.d  n.d |
| Stensvold et al. 2010 [[21](#_ENREF_21)]  No significant differences between groups for any baseline variable | AET: 99.7±18.7  RT: 103.4±17.1  CT: 92.1±14.8 | AET: 109.6±10  RT: 111.5±10.8  CT: 106.2±8.6 | n.d  n.d  n.d | AET: 33.5±8.6  RT: 32.3±7  CT: 32.8±4.6 | AET: 63.5±15.2  RT: 68.2±12  CT: 56.3±12.3 | AET: 235.52±37.06  RT: 211.96±46.71  CT: 243.24±33.97 | n.d  n.d  n.d | AET: 45.17±13.51  RT: 44.4±6.94  CT: 53.28±14.67 | AET: 203.72±88.57  RT: 159.43±79.71  CT: 230.29±124 | AET: 34.2±9.8  RT: 31.9±4.6  CT: 28.4±6.3 |
| Wallace et al. 1997 [[20](#_ENREF_20)]  No significant differences between groups for any baseline variable | AET: 93.3 ±15.1  CT: 94.65±12.24 | n.d  n.d | AET: 1.05±0.08  CT: 1.02±0.08 | AET: 25.98 ±8.34  CT: 25.76±6.98 | AET: 67.18 ±7.52  CT: 64.56±22.65 | n.f  n.d | n.d  n.d | AET: 32.25±4.52  RT: 30.75±4.21 | AET: 191.13±37.5  RT: 206.5±63.35 | AET: 27.03 ±3.5  CT: 25.41±2.99 |

AET, aerobic endurance training; BW, body weight; CT, combined training (RT and AET); Ex, exercises; FM, fat mass; HDL-C, high-density lipoprotein cholesterol; LBM, lean body mass; LDL-C, low-density lipoprotein cholesterol; n.d, no data; R, Repetition; RT, resistance training; TC, total cholesterol; TG, triacylglycerols; VO_2_ max, maximal oxygen uptake; WC, waist circumference; WHR, waist to hip ratio; *reported as interquartile range.
